# VCAM-1 in Cognitive Impairment: Mechanisms, Biomarker Potential, and Therapeutic Targeting

**DOI:** 10.14336/AD.2025.0675

**Published:** 2026-07-09

**Authors:** Xilong Guan, Xiaoling Zhu, Linan Zha, Wen Yu, Xiuqin Rao, Yanhong Xiong, Yu Jin, Mojiao Zhang, Tao Luo, Xiangfei Huang, Xifeng Wang, Fuzhou Hua, Jing Xu

**Affiliations:** ^1^Department of Anesthesiology, Yingtan City People’s Hosptial, Yingtan, Jiangxi, China.; ^2^Key Laboratory of Anesthesiology of Jiangxi Province, Nanchang, Jiangxi, China.; ^3^Department of Anesthesiology, the First Affiliated Hospital. Jiangxi medical College, Nanchang University, Nanchang, Jiangxi, China.; ^4^Department of Anesthesiology, the Second Affiliated Hospital. Jiangxi medical College, Nanchang University, Nanchang, Jiangxi, China.

**Keywords:** VCAM-1, cognitive impairment, BBB, neuroinflammation, biomarkers, therapeutic targets

## Abstract

Cognitive impairment (CI), a progressive decline in memory, reasoning, and executive functions, arises from neurodegenerative and cerebrovascular pathologies. Globally, 10% of adults aged ≥65 exhibit mild CI (MCI), with 15% progressing annually to dementia. Alzheimer’s disease (AD) constitutes 60-70% of dementia cases, showing a higher incidence in women, while vascular dementia (VaD) accounts for 20%. By 2050, dementia cases may reach 152 million, straining healthcare systems. Vascular cell adhesion molecule 1 (VCAM-1), a key immune-inflammatory mediator, is implicated in CI pathogenesis. Expressed abundantly on endothelial cells, VCAM-1 disrupts blood-brain barrier (BBB) integrity and exacerbates neuroinflammation. This review delineates VCAM-1’s role in BBB dysregulation, neuroinflammatory interactions, and its potential as a biomarker/therapeutic target. Future research should clarify VCAM-1 signaling mechanisms and develop targeted interventions for early CI management.

## 1. Introduction

Cognitive impairment, a significant public health issue worldwide, covers a range of disorders such as Alzheimer's disease (AD), vascular cognitive impairment (VCI), and multiple types of dementia. Due to global population aging, the number of dementia patients worldwide has exceeded 55 million, with AD being the most prevalent form [1-3]. These disorders not only severely impair patients' daily functioning and quality of life but also impose a substantial social and economic burden. Emerging research suggests that these conditions share common pathogenic mechanisms, particularly neuroinflammatory responses and impairment of the blood-brain barrier (BBB) [4-11]. VCAM-1, a member of the immunoglobulin superfamily, has emerged as a critical molecular link between neuroinflammation and cognitive decline.

VCAM-1 regulates both physiological and pathological processes, including inflammation and intercellular adhesion [12-18] and is primarily expressed in activated vascular endothelial cells under physiological conditions. Through its immunoglobulin-like domain, VCAM-1 binds to ligands such as integrin α4β1 (very late antigen-4, VLA-4), thereby modulating leukocyte adhesion, migration, and BBB homeostasis [12, 13, 19, 20]. Under pathological conditions, the upregulation of VCAM-1 expression shows significant associations with the progression of cognitive disorders. For example, in AD patients, plasma VCAM-1 levels correlate positively not only with the Clinical Dementia Rating-Sum of Boxes (CDR-SB) and medial temporal lobe atrophy but also demonstrate synergistic predictive value alongside the cerebrospinal fluid Aβ42/tau index [21, 22]. Similarly, in Parkinson’s disease (PD), elevated serum VCAM-1 levels correlate with worsening motor symptom severity [23, 24]. Likewise, in VCI models, the increased cerebral endothelial VCAM-1 expression closely aligns with BBB damage severity [25]. Notably, VCAM-1’s pathogenic role extends across multiple disorders, including long-term consciousness impairment, primary progressive multiple sclerosis (PPMS), postoperative cognitive dysfunction (POCD), and HIV-associated cognitive decline [26-30].

Longitudinal data have reinforced the clinical significance of VCAM-1, demonstrating that baseline levels predict the 10-year cumulative incidence of cognitive impairment, with every 100 ng/mL increase associated with a 30% higher risk of cognitive deterioration in PPMS patients [27, 31]. Multicenter studies have established VCAM-1's significant associations with specific cognitive domains including executive function, working memory, and verbal memory, while revealing that its dynamic changes may serve as a marker for 5-year cognitive decline trajectories [32-34]. Mechanistic evidence indicates that VCAM-1 contributes to cognitive impairment through dual pathogenic pathways: promoting leukocyte transmigration across the BBB to exacerbate neuroinflammation and concurrently disrupting neuro-vascular exchange between the cerebrovascular and central nervous systems [13, 35]. These findings are further supported by in vivo studies validating VCAM-1 upregulation in the brains of AD animal models [36], collectively underscoring its central role in cognitive pathophysiology.

Despite substantial advances in understanding VCAM-1's role in cognitive impairment, critical knowledge gaps persist. Current clinical evidence remains predominantly cross-sectional, precluding definitive conclusions about causal relationships between VCAM-1 dynamics and cognitive decline progression. Furthermore, while VCAM-1 has emerged as a promising inflammatory biomarker, its limited disease specificity and subtype-dependent regulatory mechanisms in various cognitive disorders require systematic characterization. The translational potential of VCAM-1-targeted interventions also remains uncertain, as existing studies are largely confined to preclinical models. Addressing these limitations will necessitate multidisciplinary approaches combining multi-omics profiling to decipher VCAM-1's regulatory networks with advanced in vivo imaging technologies capable of tracking its spatiotemporal contributions to neuropathology [33, 37-42]. Such integrated strategies may accelerate the development of VCAM-1-based diagnostics and therapeutics for cognitive disorders.

## 2. Overview of VCAM-1

### 2.1 Structure and function

VCAM-1 (CD106), a 110 kDa transmembrane glycoprotein belonging to the immunoglobulin superfamily, mediates leukocyte-endothelial adhesion through its extracellular domain [19, 43, 44], with constitutive expression observed predominantly in vascular endothelial cells [45]. VCAM-1 was initially characterized in 1989 as a glycoprotein expressed on endothelial cell surfaces [46]. VCAM-1 expression is upregulated through multiple pathways, including cytokine signaling such as tumor necrosis factor-α (TNF-α), metabolic dysregulation including hyperglycemia and oxidized LDL, mechanical stress from shear forces, and pattern recognition receptor activation by Toll-like receptor agonists [14, 47, 48]. Under pathological conditions spanning acute inflammation to chronic disease states, VCAM-1 expression extends beyond endothelial cells to include immunocompetent cells (macrophages, dendritic cells), stromal elements (fibroblasts, myofibroblasts), specialized parenchymal cells (Kupffer cells, Sertoli cells), and neoplastic populations [49]. Accumulating evidence implicates VCAM-1 in the pathogenesis of diverse clinical entities ranging from oncogenesis and metastatic progression to cardiovascular (atherosclerosis), rheumatological (arthritis), and transplantation-related pathologies [50-54].

**Figure 1.**
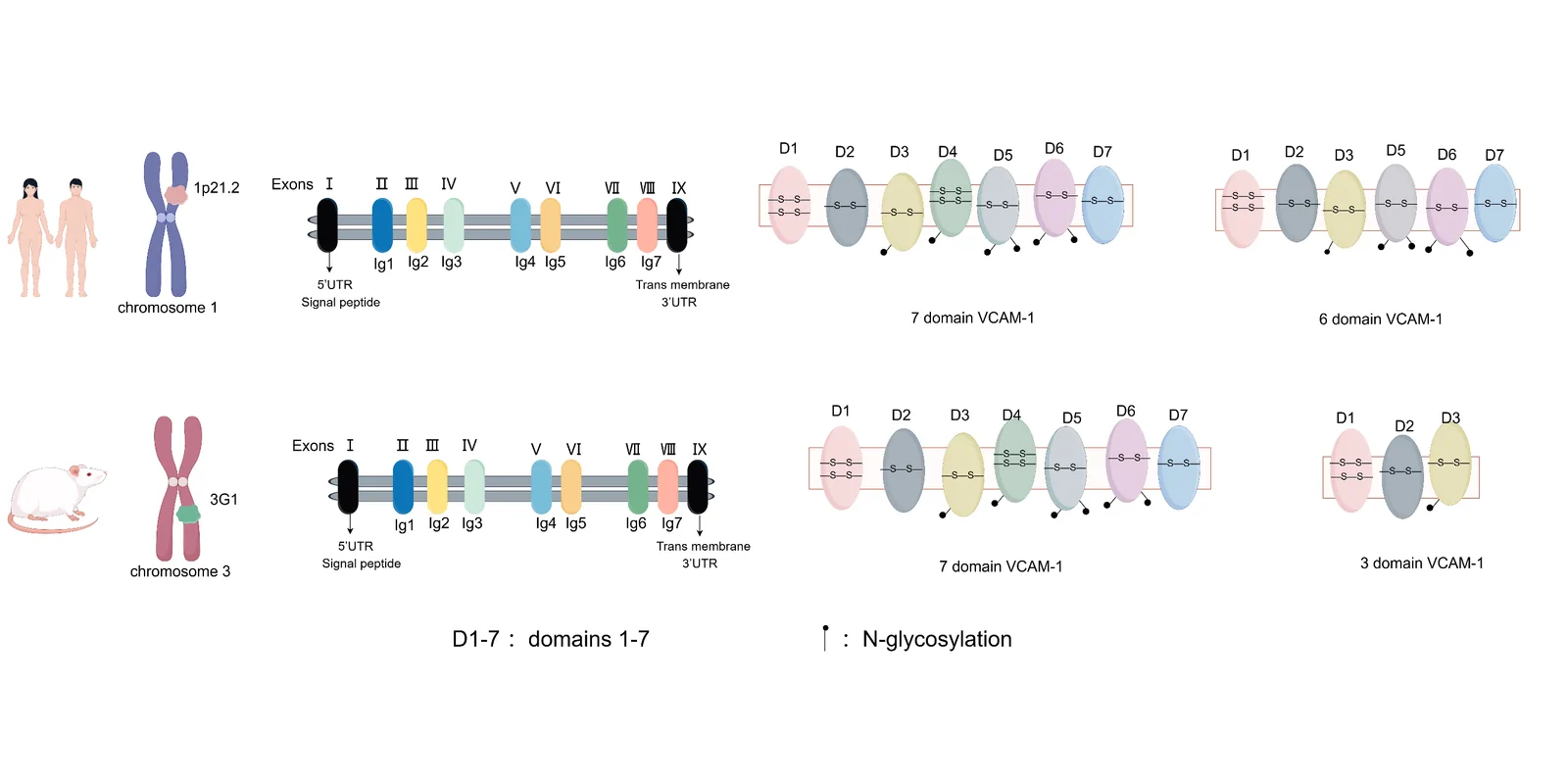
**Molecular Structures of VCAM-1 in Human and Mouse**. In humans, VCAM-1 is a protein featuring an extracellular domain composed of six or seven immunoglobulin (Ig)-like domains. These domains contain disulfide-bonded loops and N-linked glycosylation sites that facilitate interactions with galectin-3 on eosinophils. The gene encoding human VCAM-1 is situated on chromosome 1 at locus 1p21.2 and comprises nine exons. These exons span various functional regions, such as the 5' untranslated region (5' UTR), signal peptide sequence, transmembrane domain, and 3' untranslated region (3' UTR). In mice, the VCAM-1 protein structure differs slightly, containing either three or seven Ig-like domains. The murine VCAM-1 gene is located on chromosome 3 in the 361 region and also consists of nine exons, mirroring the organizational pattern seen in humans.

As a type I transmembrane glycoprotein, VCAM-1 exhibits conformational flexibility through its immunoglobulin (Ig)-type extracellular domains [55]. The canonical human VCAM-1 isoform contains seven extracellular Ig-like domains (D1-D7) organized in tandem, connected via disulfide-stabilized loops, with D1 and D4 containing critical N-glycosylation sites that mediate galectin-3 interactions on eosinophils [56]. These structural domains facilitate binding to galectin-3 through disulfide-bonded loops and N-glycosylation sites [56, 57]. VCAM-1's Ig-like domains 1 and 4 are primarily involved in ligand binding [58], particularly with α4β1 and α4β7 integrins [59]. The α4β1 integrin-VCAM-1 interaction orchestrates leukocyte recruitment through sequential biomechanical events, beginning with initial tethering under shear stress, followed by adhesion stabilization via inside-out signaling, and culminating in transendothelial migration guided by chemokine gradients [55, 60]. The human VCAM-1 gene spans 25 kb at chromosomal locus 1p21.2 and contains nine exons encoding a 43-nucleotide 5' untranslated region (UTR), signal peptide (exon 2), extracellular domains (exons 3-7), transmembrane segment (exon 8), and 3' UTR (exon 9). In contrast to the human form, murine VCAM-1 exhibits a distinct structural organization, featuring only three extracellular Ig domains (homologous to human D1-D3) and four membrane-associated domains. Despite this structural divergence, the mouse Vcam1 gene maintains conserved synteny on chromosome 3 (3G1) with analogous exon-intron architecture [61]. Evolutionary analysis reveals >85% sequence homology in integrin-binding domains across species, preserving VCAM-1's core biological functions and maintaining cross-species ligand recognition capacity [62] (Fig. 1).

### 2.2 Differential analysis of VCAM-1 and other endothelial biomarkers in CI diagnosis

In the context of CI, endothelial biomarkers play a crucial role in reflecting pathological mechanisms, including BBB damage, intracranial inflammation, and microthrombus formation. As adhesion molecules, VCAM-1 and intercellular adhesion molecule-1 (ICAM-1) can mediate the trans-BBB migration of inflammatory cells. In AD and vascular dementia (VaD), the levels of these molecules in serum and cerebrospinal fluid are closely related to the severity of neuroinflammation and the rate of cognitive decline. When used in combination with neurodegenerative markers such as amyloid-β (Aβ) and tau protein, they can improve the diagnostic accuracy of AD [21, 63].

E-selectin is rapidly upregulated after acute cerebral ischemia and is a sensitive marker for the early identification of ischemic cognitive impairment, but its clinical application needs to be combined with imaging examinations to exclude other etiologies [64, 65].

Meanwhile, von Willebrand factor (vWF) affects cerebral blood flow perfusion by promoting microthrombus formation, and soluble thrombomodulin (sTM) does so by disrupting the intracranial anticoagulant balance [66]. In cerebral small vessel disease, the levels of both are positively correlated with microthrombus burden and BBB permeability [67], which is of great value in evaluating the progression of VaD and guiding antiplatelet therapeutic strategies [68].

These biomarkers are interrelated through the pathological cascade of "inflammation-endothelial injury-cerebral perfusion." The combined detection of them can analyze the pathogenesis of CI from multiple dimensions, providing a scientific basis for the accurate differentiation between AD and VaD, the prediction of the risk of cognitive decline, and the formulation of targeted treatment plans [69].

As shown in Table 1, VCAM-1 has high diagnostic accuracy (AUC values ranging from 0.78 to 0.85) in the diagnosis of AD and VaD, with high sensitivity (70%-80%) and specificity (75%-85%). Compared with other biomarkers, VCAM-1 shows broader application potential in the diagnosis of various types of cognitive impairment. For example, although ICAM-1 also has certain diagnostic capabilities, its sensitivity and specificity are slightly lower than those of VCAM-1. In addition, E-selectin is mainly applicable to the early identification of ischemic cognitive impairment, while vWF and sTM show certain value in the diagnosis of VaD. Despite the advantage of VCAM-1 in diagnostic accuracy, its detection process still faces challenges, such as the impact of sample type and processing time on detection results. Therefore, future research needs to further optimize detection methods, establish standardized detection procedures, and verify its clinical application value through multicenter large-sample studies.

**Table 1:** Comparative Analysis of Biomarkers in CI Diagnosis.

Biomarker	Diagnostic Accuracy (AUC)	Sensitivity (%)	Specificity (%)	Applicable Disease Types
**VCAM-1**	0.78-0.85	70-80	75-85	AD, VD
**ICAM-1**	0.72-0.76	65-75	70-80	AD, VD
**E-selectin**	0.68-0.70	60-70	65-75	Ischemic cognitive impairment
**vWF**	0.65-0.70	55-65	60-70	VD
**sTM**	0.67-0.72	60-70	65-75	VD

Notes: Diagnostic Accuracy (AUC), Represents the overall ability of the biomarker to distinguish between patients and healthy controls. An AUC value closer to 1 indicates stronger diagnostic capability; Sensitivity, Indicates the biomarker's ability to correctly identify patients, i.e., the true positive rate; Specificity, Indicates the biomarker's ability to correctly exclude non-patients, i.e., the true negative rate; Applicable Disease Types: Lists the types of cognitive impairment in which the biomarker shows better diagnostic performance.

### 2.3 The role in the BBB

The BBB constitutes a selective interface between the systemic circulation and the central nervous system (CNS) [70], composed of cerebral microvascular endothelial cells, tight junctions, astrocytes, pericytes, and basement membranes [71-75]. These components collectively restrict the paracellular movement of substances. The primary role of the BBB is to orchestrate molecular flux between the bloodstream and the brain, safeguarding the brain from detrimental substances (endogenous or exogenous) [76-78], while permitting nutrient penetration [79]. It is vital for preserving CNS homeostatic equilibrium [80]. Moreover, the BBB actively eliminates certain drugs and toxic compounds through efflux transporters, including P-glycoprotein and breast cancer resistance proteins [81], preserving brain homeostasis and preventing brain damage [82]. Consequently, BBB functional and structural integrity is essential for maintaining microenvironmental homeostasis in the brain [80].

VCAM-1 exerts multivalent regulatory functions within the neurovascular unit [23], maintaining constitutive low-level expression on quiescent BBB endothelium under homeostatic conditions [83]. As a multifunctional mediator, it orchestrates cell-cell interactions and modulates signaling pathways, serving as a crucial regulator of basal BBB integrity [83] and stabilizing BBB cytoarchitectural and functional connections via specific cell surface molecule interactions. The role of VCAM-1 is particularly significant during pathological processes. During episodes of brain inflammation, whether caused by infections, autoimmune disorders, or other pathological conditions, various inflammatory factors can increase the expression of VCAM-1 on the endothelial cells of the BBB and associated cells. This heightened expression may contribute to BBB impairment [84, 85], permitting increased immune cell influx into the CNS and exacerbating neuroinflammation and neuronal damage.

### 2.4 The relationship with neuroinflammation

Neuroinflammation represents a stereotypic glial response marked by microglial activation and astrogliosis, induced through multiple pathogenic mechanisms including pathogen-associated molecular patterns such as LPS, damage-associated molecular patterns including HMGB1, metabolic disturbances like vitamin B12 deficiency, and autoantibody-mediated immune responses [86]. This condition markedly impacts nervous system structure and function, leading to various neurological disorders [87-89]. These effects severely compromise patients' quality of life and, in severe cases, threaten survival.

As an inflammation-associated adhesion molecule, VCAM-1 is a key neuroinflammatory mediator, participating in both inflammatory processes/nerve injury [90] and neural development/repair [21]. Following neural tissue injury or infection, pathogenic factors induce VCAM-1 overexpression, promoting leukocyte migration into the CNS [55, 91, 92]. Infiltrating leukocytes release inflammatory mediators, perpetuating neuro-inflammation. Accumulating evidence indicates that neuroinflammation contributes significantly to cognitive impairment pathogenesis [5], where persistent inflammation induces neuronal damage, disrupts neural signaling, impairs synaptic plasticity, and ultimately compromises cognitive function. Interestingly, during neuroinflammation resolution, VCAM-1 facilitates neural repair. Emerging evidence shows VCAM-1-dependent controlled immune cell infiltration promotes necrotic tissue clearance and neuroregenerative factor secretion [93].

**Figure 2.**
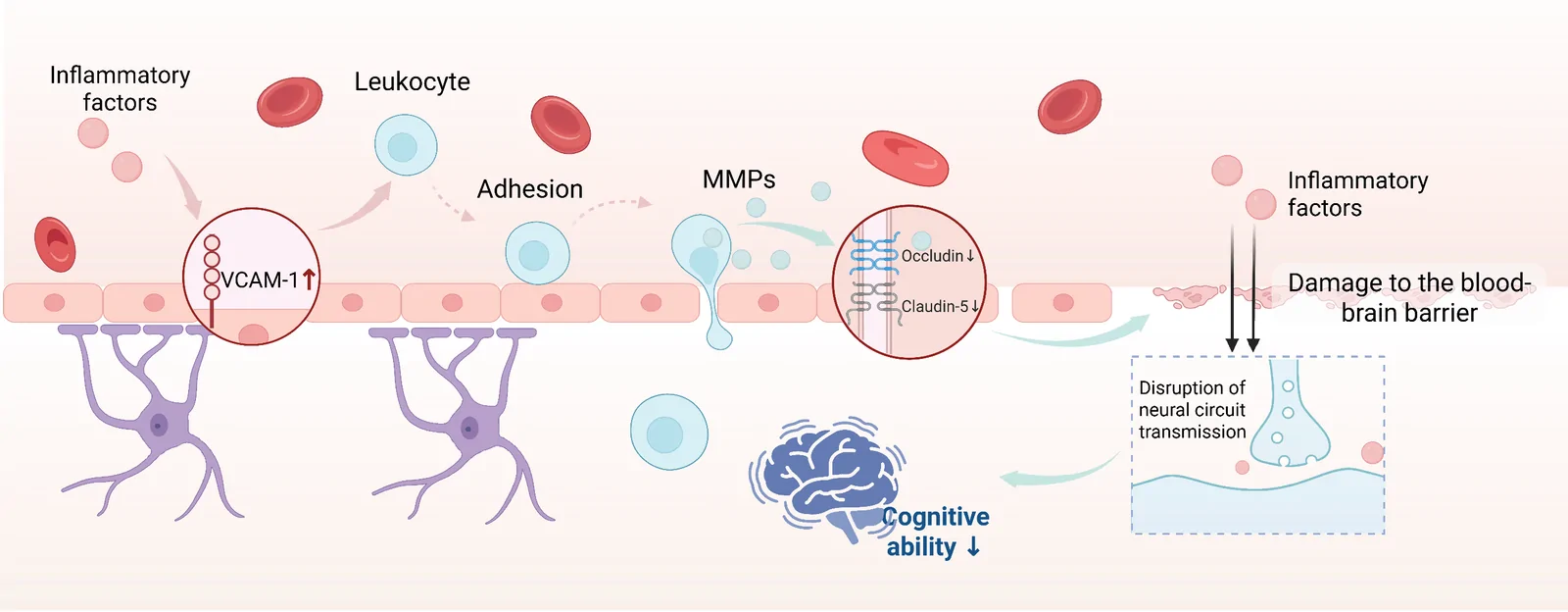
**Mechanistic Diagram of VCAM-1-Mediated Cognitive Impairment via BBB-Neuroinflammation Mechanism**. When inflammatory factors such as TNF-α and IL-1β stimulate cerebral vascular endothelial cells, the expression of VCAM-1 significantly increases. This elevated VCAM-1 expression enhances the adhesion of specific white blood cells, including monocytes and lymphocytes, to cerebral vascular endothelial cells. These adhered white blood cells will then pass through the endothelial cell gap or induce endothelial cell damage, thereby disrupting the tight junction structure and damaging the integrity of the BBB. After the integrity of the BBB is disrupted, inflammatory mediators and neurotoxic substances in the blood can enter the brain, activating microglia. Overactivated microglia will release more inflammatory mediators, such as IL-6 and IL-1β, triggering neuroinflammatory reactions and affecting cognitive function.

## 3. Mechanisms by which VCAM-1 affects cognitive impairment

### 3.1 BBB-neuroinflammation and immune cell infiltration cascade

Recent investigations have underscored the role of VCAM-1 in cognitive impairment through interlinked pathological pathways [24, 30, 33, 80, 94-103]. Within the BBB-neuroinflammation axis, VCAM-1 dysregulation compromises BBB integrity: proinflammatory cytokines including TNF-α and interleukin-1β (IL-1β) induce VCAM-1 upregulation in cerebral endothelial cells, promoting adhesion of monocytes/lymphocytes that secrete matrix metalloproteinases (MMPs) to degrade tight junction proteins such as occludin and claudin-5 [76-78, 104-106].

**Figure 3.**
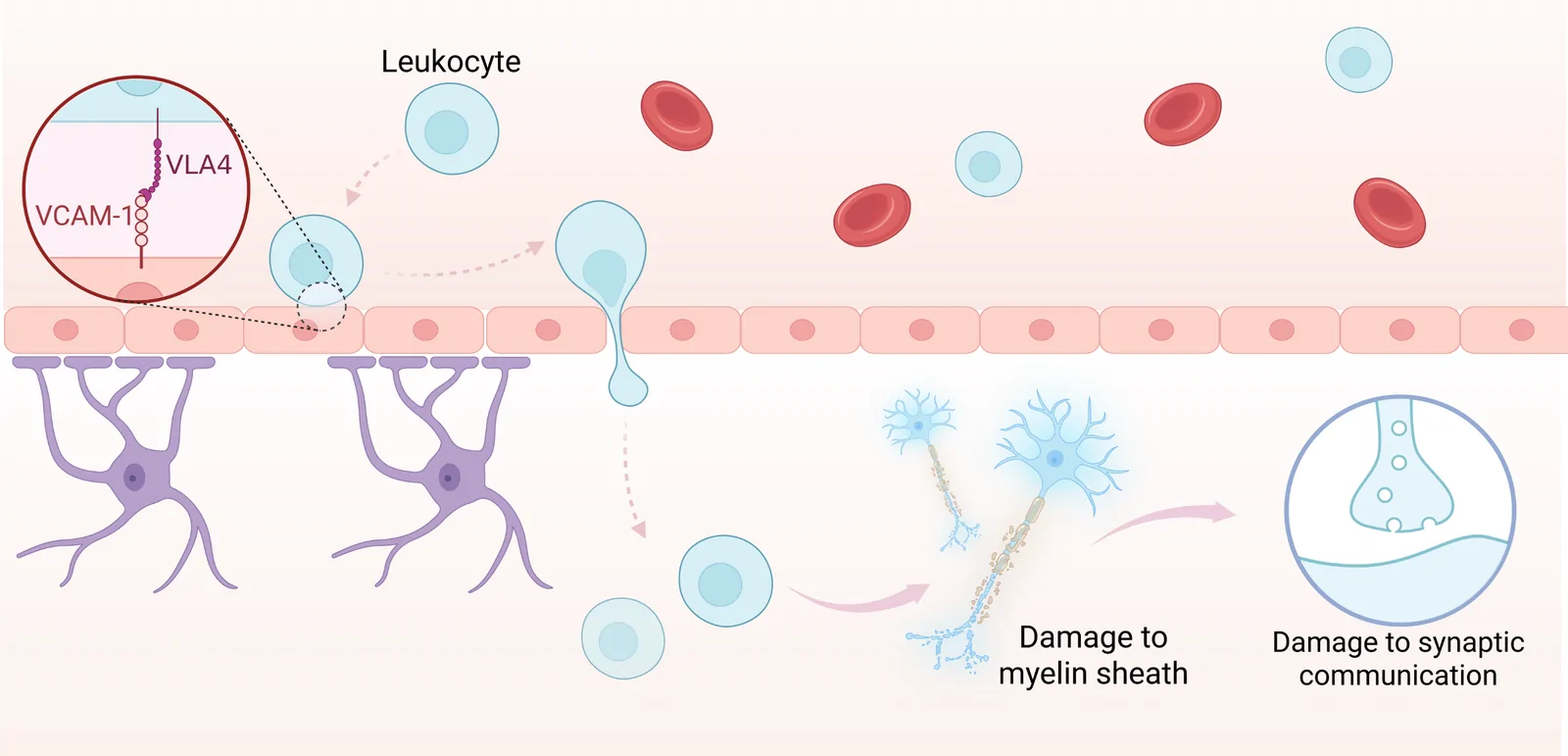
**Mechanistic Diagram of VCAM-1-Mediated Cognitive Impairment via Immune Cell Infiltration**. VCAM-1 interacts with specific ligands including VLA-4 to mediate the transmigration of immune cells such as leukocytes across the BBB. These infiltrating cells target neural cells in the CNS, leading to demyelination and consequent impairment of cognitive function.

This enhances BBB permeability, enabling neurotoxins and inflammatory mediators to infiltrate the brain parenchyma, activate microglia, and trigger a proinflammatory cascade [107-111]. In cognitive-critical regions like the hippocampus, this impairs neuronal excitability and synaptic plasticity, manifesting as memory deficits. In AD, Aβ deposition further upregulates VCAM-1, amplifying neuroinflammation to promote tau hyperphosphorylation and neuronal dysfunction [112] (Fig. 2).

As a member of the immunoglobulin superfamily, VCAM-1 mediates leukocyte transmigration across the BBB via integrin VLA-4, a mechanism observed in multiple sclerosis and AD [81, 113-121]. Infiltrating immune cells such as T lymphocytes damage oligodendrocytes, causing myelin degradation that disrupts saltatory conduction and functional connectivity within networks like the default mode network (DMN) [122-125]. This leads to clinical impairments in memory, attention, and executive function (Fig. 3).

### 3.2 Cerebrovascular dysfunction and BBB homeostasis disruption

**Figure 4.**
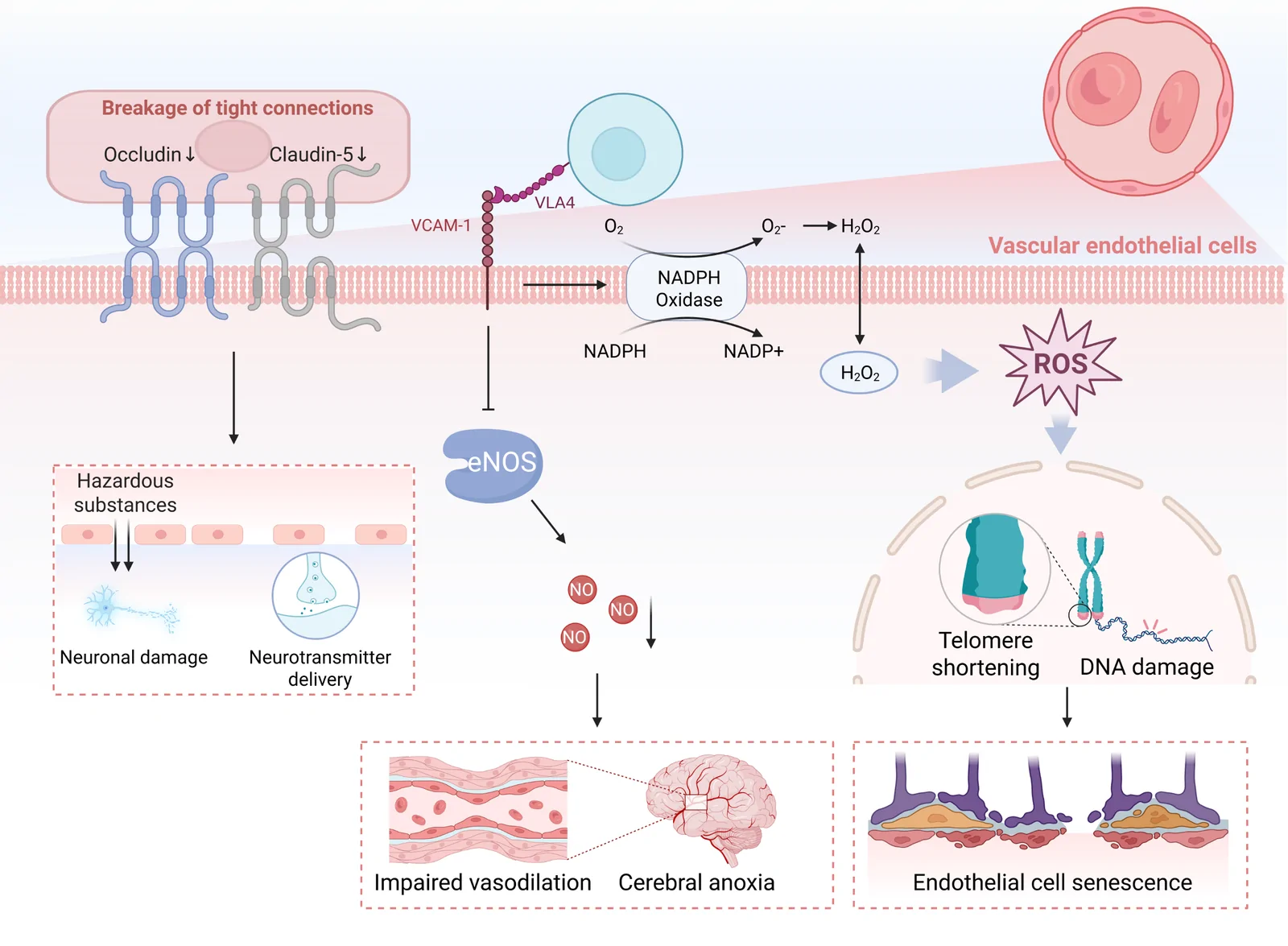
**Mechanistic Diagram of VCAM-1-Mediated Cognitive Impairment via Cerebrovascular Dysfunction**. Increased VCAM-1 expression activates endothelial cell signaling pathways that inhibit eNOS activity, reducing NO production and impairing cerebral vasodilation. This decreases cerebral blood flow, ultimately compromising cognitive function. VCAM-1-associated inflammatory signaling downregulates tight junction protein expression and disrupts their structure, increasing BBB permeability. This BBB dysfunction permits neurotoxins to infiltrate brain tissue, causing neuronal damage. Additionally, it destabilizes the cerebral microenvironment, impairing neurotransmitter and nutrient transport, which further contributes to cognitive dysfunction.

VCAM-1 further contributes to cerebrovascular dysfunction by promoting endothelial cell apoptosis through NADPH oxidase-mediated reactive oxygen species (ROS) generation and suppressing endothelial nitric oxide synthase (eNOS) activity, thereby reducing nitric oxide (NO) production and impairing vascular dilation [126-134]. Concurrently, VCAM-1 disrupts BBB tight junctions via inflammatory signaling pathways, exacerbating BBB permeability and microenvironmental instability [135]. In VCI, this establishes a pathological vicious cycle with cerebrovascular lesions, thereby exacerbating cognitive deficits [24] (Fig. 4).

### 3.3 Dysregulation of amyloid metabolism in AD

**Figure 5.**
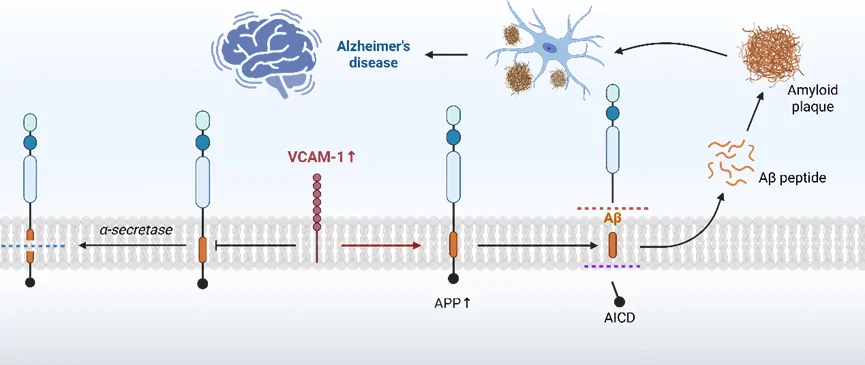
**Mechanistic Diagram of VCAM-1-Mediated Cognitive Impairment via Dysregulated Amyloid-β Metabolism**. Elevated VCAM-1 levels activate Aβ synthesis pathways while simultaneously suppressing Aβ degradation mechanisms. Aberrant VCAM-1 expression alters APP levels, consequently impairing cognitive function.

Regarding amyloid metabolism, dysregulated VCAM-1 in AD augments Aβ synthesis and suppresses its clearance by modulating amyloid precursor protein (APP) processing [111]. In transgenic animal models, VCAM-1 overexpression correlates with heightened Aβ deposition, whereas its inhibition reduces Aβ burden, underscoring its role in disrupting Aβ homeostasis (Fig. 5).

## 4. Differential pathological roles in neurodegenerative and vascular cognitive impairment

VCAM-1 exerts a pivotal pathogenic role in cognitive impairment disorders (including AD and VaD) by mediating inflammatory cell infiltration and BBB disruption. Nevertheless, its pathophysiological mechanisms demonstrate a marked dichotomy between neurodegenerative and cerebrovascular etiologies.

In AD, VCAM-1 modulates microglial chemotaxis and Aβ clearance via the VCAM-1-ApoE signaling axis. A study by Ye Yuru’s group revealed that IL-33 induces VCAM-1 upregulation in microglia, promoting their migration toward Aβ plaques and augmenting Aβ phagocytosis. Disruption of the VCAM-1-ApoE interaction impairs this cascade, exacerbating AD neuropathology. Additionally, elevated cerebrospinal fluid levels of soluble VCAM-1 (sVCAM-1) in AD patients correlate with microglial dysfunction, indicating that dysregulated VCAM-1 signaling may potentiate AD progression [136].

In contrast, in VCI or vascular dementia (VD), the role of VCAM-1 predominantly involves endothelial inflammatory responses and ischemic injury. Clinical investigations have demonstrated that serum VCAM-1 concentrations in patients with ischemic cerebral small vessel disease (ICSVD) correlate positively with cognitive impairment severity, with elevated VCAM-1 levels potentially reflecting endothelial dysfunction and chronic inflammatory states. Furthermore, atherosclerosis -associated exosomes impact microglial metabolic homeostasis via the miR-101-3p-Nrf2 signaling axis, exacerbating white matter injury. VCAM-1 may mediate vascular neuroinflammation crosstalk in this pathological cascade [137].

## 5. The potential as a biomarker

The strong association between VCAM-1 levels and cognitive impairment has positioned it as a promising biomarker candidate. Plasma VCAM-1 measurement offers notable clinical advantages, including non-invasiveness and procedural simplicity, facilitating its potential integration into routine practice [138]. Importantly, accumulating evidence demonstrates that VCAM-1 expressions exhibit sufficient specificity and sensitivity to distinguish not only cognitively healthy individuals from those with impairment, but also different subtypes and stages of cognitive dysfunction. For example, in PD patients, Zheng et al. [36] reported that elevated serum VCAM-1 levels correlated positively with both motor symptom severity (MDS-UPDRS scores) and disease progression (Hoehn and Yahr staging), underscoring its biomarker potential. Similarly, Pietronigro et al. [121] identified increased soluble VCAM-1 in AD patients, with levels associated with advanced dementia and white matter hyperintensities, suggesting VCAM-1's role in AD-related vascular inflammation and cognitive decline. Furthermore, significantly raised VCAM-1 levels in pDoC patients highlight its diagnostic utility across neurological conditions [26]. Although VCAM-1 has demonstrated clinical utility as a biomarker for diagnosing and monitoring neuroinflammatory and cognitive impairment disorders, its detection process remains fraught with challenges. Concerning assay variability, a multicenter study by de Kroon et al. [139] revealed that when detecting VCAM-1 via ELISA kits across different laboratories, interlaboratory deviation for identical specimens can reach 20%-40% owing to differences in antibody specificity—such as variations in recognized epitopes. Rogatsky et al. [140] evaluated a commercial ELISA kit and found that while its detection limit for recombinant VCAM-1 was 12.5 pg/mL, the recovery rate for native plasma samples ranged from only 82% to 115%. Such systematic errors are prone to amplification in large-scale clinical investigations.

The choice of sample type further complicates testing standardization. Amin Nordin et al. [141] demonstrated that platelet activation during serum collection—due to coagulation reactions—releases granular-stored VCAM-1, causing serum concentrations to be 18.7% higher than those in plasma. Dodig et al.[142] observed that VCAM-1 levels in heparin-anticoagulated plasma were 9.3% lower than those in EDTA-anticoagulated plasma, attributed to heparin-mediated disruption of the stability of VCAM-1-lipoprotein complexes. The timeliness of sample processing is also crucial. Sample processing timeliness is equally critical: Duque-Afonso et al. [143] confirmed that plasma samples left at room temperature for more than 2 hours exhibit a 15%-20% elevation in VCAM-1 levels due to leukocyte activation, while repeated freeze-thaw cycles induce protein degradation, reducing detection values by more than 30%.

Currently, no unified standard exists for determining cutoff values in research. Li et al. [144] established through case-control studies that the cerebrospinal fluid VCAM-1 cutoff for AD diagnosis is 245 pg/mL (AUC=0.78), while Sun et al. [145] reported a plasma VCAM-1 cutoff of 876 ng/L (AUC=0.69) in patients with vascular cognitive impairment. Physiological factors profoundly influence threshold setting: Huang et al. [146] observed that healthy elderly populations exhibit 42% higher VCAM-1 levels than younger groups, and females show 21% lower levels than males due to estrogen regulation. Comorbidities further complicate interpretation: glycative stress in diabetic patients increases plasma VCAM-1 by 2.3-fold, while reduced renal clearance in chronic kidney disease (CKD) elevates VCAM-1 by 1.8-fold. Such findings highlight that a single cutoff value is inadequate for the nuanced diagnostic demands of complex clinical scenarios [147].

To tackle the aforementioned challenges, the establishment of a standardized testing system is imperative. Chen et al. [148] demonstrated the potential of data correction by improving the area under the curve (AUC) for VCAM-1 in Alzheimer’s disease diagnosis from 0.72 to 0.85 using a machine learning correction model. Studies have shown that standardization of biomarker testing is pivotal for clinical decision-making, as failure to account for assay variability may lead to a 25-30% misdiagnosis rate. Future research should prioritize optimizing detection methodologies, standardizing sample processing protocols, and defining more precise cutoff values through multicenter large-sample studies to facilitate the translation of VCAM-1 from a laboratory indicator to a reliable clinical biomarker.

The ATNIVS model provides a comprehensive framework for understanding the multifactorial nature of cognitive impairment. As shown in Table 2, each component of the ATNIVS model is associated with specific biomarkers that can be measured in clinical settings. For instance, Aβ and tau biomarkers are critical for early detection and differentiation of AD from other forms of dementia. Neurodegenerative markers such as neurofilament light chain (NfL) and FDG-PET can help identify ongoing neurodegenerative processes and predict cognitive decline. Inflammation-related biomarkers like VCAM-1, ICAM-1, and E-selectin are essential for assessing neuroinflammation and identifying vascular contributions to cognitive impairment. Vascular biomarkers such as vWF, sTM, and VCAM-1 can evaluate cerebrovascular health and predict the risk of vascular dementia. Finally, synaptic biomarkers like SV2A can monitor synaptic dysfunction and serve as potential therapeutic targets.

Integrating these biomarkers into clinical practice can enhance diagnostic accuracy, facilitate early intervention, and guide personalized treatment strategies. For example, combining Aβ and tau biomarkers with neuro-inflammatory markers can provide a more comprehensive understanding of disease progression and help tailor treatments that address both neurodegenerative and inflammatory components. Future research should focus on validating these biomarkers in diverse populations and developing standardized protocols for their measurement and interpretation. This approach will not only improve the accuracy of diagnosis but also aid in the development of targeted therapies that can effectively manage the various components of cognitive impairment.

By incorporating such modifications, you can better address the reviewer's comments and provide readers with a more comprehensive and clinically relevant understanding of the ATNIVS model and its biomarkers.

**Table 2:** Mapping of Biomarkers to the ATNIVS Model Components.

Component of ATNIVS Model	Related Biomarkers	Clinical Implications
**Aβ (Amyloid)**	Plasma Aβ42, CSF Aβ42	Early detection of Alzheimer's disease; monitoring disease progression
**T (Tau)**	CSF total tau, CSF phosphorylated tau	Differentiating Alzheimer's disease from other dementias; assessing severity
**N (Neurodegeneration)**	Neurofilament light chain (NfL), FDG-PET	Identifying neurodegenerative processes; predicting cognitive decline
**I (Inflammation)**	VCAM-1, ICAM-1, E-selectin	Assessing neuroinflammation; identifying vascular contributions to cognitive impairment
**V (Vascular)**	vWF, sTM, VCAM-1	Evaluating cerebrovascular health; predicting risk of vascular dementia
**S (Synaptic)**	Synaptic vesicle glycoprotein 2A (SV2A)	Monitoring synaptic dysfunction; potential therapeutic target

Notes: Component of ATNIVS Model, the components of the ATNIVS model, which stands for Amyloid, Tau, Neurodegeneration, Inflammation, Vascular, and Synaptic; Related Biomarkers, Biomarkers that are associated with each component of the ATNIVS model; Clinical Implications, The potential clinical applications of these biomarkers in the diagnosis, monitoring, and treatment of cognitive impairment.

## 6. The potential as a therapeutic target

In the therapeutic research of neuroinflammation and cognitive impairment, therapeutic approaches targeting VCAM-1, especially antibody-based interventions and drug blockades, have shown promising prospects. Monoclonal antibodies against VCAM-1, with high specificity, can precisely bind to VCAM-1, block its interaction with ligands such as VLA-4, effectively inhibit leukocyte adhesion and transendothelial migration, and thus suppress the neuroinflammatory cascade [20, 113, 149]. With advancements in antibody development technologies, the potential applications of these therapeutic strategies in diagnostic imaging and pathological intervention are continuously expanding [150]. Preclinical data robustly validate their efficacy, as exemplified by Yousef et al. [151], who found that anti-VCAM-1 antibody therapy in senescent mice augments neural progenitor cell activity, mitigates microglial activation, and significantly ameliorates hippocampus-dependent learning and memory functions. However, monoclonal antibody (mAb) therapy faces multifaceted challenges during clinical translation. Pharmacokinetically, mAbs exhibit high molecular weight, low plasma clearance rates, and prolonged systemic circulation times. While this profile facilitates sustained therapeutic concentration, it may also elevate the risk of adverse reactions. Concomitantly, the BBB imposes a permeability barrier to mAbs, limiting CNS penetrance and resulting in suboptimal drug concentrations in the CNS, which hinder full therapeutic efficacy. Immunogenicity is a critical challenge, as antibody-based interventions readily elicit host immune responses—particularly in chronic administration regimens, where anti-drug antibodies (ADA) may develop, compromising therapeutic efficacy and potentially inducing severe adverse events that threaten treatment safety.

In contrast, small-molecule therapeutics demonstrate efficacy in mitigating neuroinflammation and restoring BBB integrity by inhibiting VCAM-1 expression or disrupting its signaling axis, thereby downregulating pro-inflammatory gene transcription and holding promise for therapeutic breakthroughs in cognitive impairment [152]. Yet small-molecule inhibitors are not without limitations: their therapeutic specificity challenges precise modulation, potentially leading to off-target effects on essential physiological pathways and compromising normal organ functions. Rigorous preclinical evaluation of small-molecule candidates is therefore imperative during drug design and development to minimize off-target risks.

New research evidence continuously reinforces the theoretical foundation of VCAM-1 as a therapeutic target for cognitive impairment. Preclinical investigations have demonstrated that modulation of the VCAM-1 signaling axis effectively ameliorates neurodegenerative and cerebrovascular pathologies. A study by the Hong Kong University of Science and Technology has revealed the ameliorative effect of VCAM-1-APOE pathway activation on AD pathology in animal models, providing critical mechanistic support for VCAM-1-targeted intervention [136]. Research in VCI models also indicates that VCAM-1 inhibition holds potential for improving cerebrovascular function in comorbidities like atherosclerosis and diabetes mellitus [153]. However, current research still has numerous unresolved questions, including the precise molecular mechanisms by which VCAM-1 contributes to VCI pathogenesis, the optimal regulatory strategies for antibody-based and small-molecule inhibitors, and the potential off-target effects of systemic VCAM-1 inhibition. Future studies should prioritize the development of targeted therapeutics capable of accurately and selectively modulating VCAM-1 activity, thereby minimizing adverse impacts on physiological vascular function.

Although VCAM-1-targeted therapy has demonstrated promising outcomes in preclinical investigations, it must overcome numerous challenges before clinical translation [149]. Beyond the immunogenicity of monoclonal antibodies and off-target effects of small-molecule inhibitors discussed earlier, clinical translation is also hindered by model limitations. Existing animal models struggle to fully recapitulate the complex, multifactorial pathogenesis of human cognitive impairment, introducing substantial uncertainty in predicting clinical outcomes. To advance research, there is an urgent need to refine antibody humanization technologies, enhance comprehensive toxicological profiling of candidate agents, and actively explore more sophisticated humanized animal models or organoid systems to better recapitulate human disease progression.

## 7. Ongoing Clinical Trials and Biomarker Validation Efforts

To contextualize future directions in the field of cognitive impairment and biomarker research, it is essential to highlight ongoing clinical trials and validation efforts. These initiatives provide valuable insights into the potential clinical applications of biomarkers such as VCAM-1 and contribute to the development of standardized diagnostic and therapeutic approaches.

### 7.1 Major Research Networks and Ongoing Clinical Trials

The Alzheimer’s Disease Neuroimaging Initiative (ADNI) and the Dominantly Inherited Alzheimer Network (DIAN) are two major research networks that have significantly advanced the understanding of AD and related dementias. ADNI, a longitudinal, multicenter study, aims to develop biomarkers for the early detection and tracking of AD by integrating imaging, genetic, and biochemical markers. Since its inception in 2004, ADNI has expanded to include various cohorts and has been instrumental in validating the use of Aβ and tau as early indicators of AD. The network also investigates blood-based markers like VCAM-1 to improve diagnostic accuracy and monitor disease progression [154, 155].

Similarly, DIAN focuses on individuals with autosomal dominant mutations that lead to early-onset Alzheimer’s disease. This international research network aims to identify early biomarkers of disease onset and progression, facilitating the development of preventive therapies. DIAN has been crucial in validating the use of Aβ and tau as early indicators of AD and continues to explore novel biomarkers, including VCAM-1, to enhance early diagnosis and intervention [156, 157].

Several clinical trials are currently underway to validate the use of VCAM-1 and other biomarkers in the context of cognitive impairment and neurodegenerative diseases. Notable trials include the A4 Study (Anti-Amyloid Treatment in Asymptomatic Alzheimer’s Disease), which investigates the efficacy of solanezumab, an anti-Aβ monoclonal antibody, in individuals with elevated brain amyloid but no cognitive impairment. The study aims to determine whether early intervention can delay cognitive decline and includes comprehensive biomarker assessments, such as VCAM-1 levels, to evaluate the impact of treatment on neuroinflammation and vascular health [158].

The DIAN-TU (DIAN Trial Unit) evaluates the safety and efficacy of multiple drugs targeting amyloid and tau in individuals with dominantly inherited Alzheimer’s disease. This trial includes comprehensive biomarker assessments to understand the mechanisms of action and potential benefits of these treatments [159]. Additionally, the EMERGE and ENGAGE Trials are Phase 3 studies evaluating the efficacy of aducanumab, an anti-Aβ monoclonal antibody, in patients with early Alzheimer’s disease. These trials assess cognitive outcomes and biomarker changes, including VCAM-1 levels, to determine the drug's impact on neuroinflammation and disease progression [160].

### 7.2 Biomarker Validation Efforts

Efforts to validate VCAM-1 as a biomarker for cognitive impairment are ongoing. Recent studies have shown that VCAM-1 levels correlate with cognitive decline and neuroinflammatory markers in AD and other neurodegenerative conditions [21,23,36]. Standardization of VCAM-1 measurement techniques and the development of reliable cutoff values are critical steps in its clinical application. Multicenter studies, such as those conducted by ADNI and DIAN, are essential for validating VCAM-1 across diverse populations and disease stages [32, 33].

## 8. Conclusion

This systematic review synthesizes current evidence on VCAM-1's multifaceted role in cognitive impairment, encompassing its structural and functional characteristics, impact on BBB integrity, contribution to neuroinflammation, epidemiological relevance, and potential as a diagnostic biomarker. As a pivotal mediator of immune and inflammatory responses, VCAM-1 critically regulates both BBB homeostasis and neuroinflammatory cascades. Substantial evidence links VCAM-1 dysregulation to cognitive decline through multiple interconnected mechanisms, including BBB integrity compromise, facilitation of immune cell transmigration, and impairment of cerebrovascular function. These pathways establish VCAM-1 as a common pathological nexus in diverse cognitive disorders, including AD and VCI, while simultaneously positioning it as a promising diagnostic biomarker. Therapeutically, VCAM-1-targeted strategies such as monoclonal antibodies and small-molecule inhibitors demonstrate potential to attenuate neuroinflammation and restore cognitive function in preclinical models. However, clinical translation requires addressing key challenges: optimizing target specificity, minimizing off-target effects, and improving translational models.

Future investigations into VCAM-1's role in cognitive impairment should prioritize large-scale validation efforts, such as coordinating VCAM-1 detection across consortia like the ADNI and the Enhancing NeuroImaging Genetics through Meta-Analysis (ENIGMA), to establish standardized cutoff values. Additionally, combination therapies should be explored, particularly testing VCAM-1 inhibitors, such as α4β1 integrin antagonists, in combination with anti-amyloid drugs in ongoing trials (NCTXXX). Furthermore, precision stratification approaches should integrate VCAM-1 into ATNIVS-based stratification tools for trial enrollment to enhance the precision of patient selection and treatment outcomes.

At the molecular level, comprehensive investigations should elucidate the precise signaling cascades and pathogenic molecular interactions, particularly VCAM-1's synergistic effects with other cytokines, receptors, and intracellular signaling molecules. Such mechanistic insights are crucial for deciphering the complex regulatory networks underlying cognitive dysfunction. Clinically, multicenter, large-scale studies are needed to validate VCAM-1's biomarker potential across diverse populations and cognitive impairment subtypes, while establishing standardized assays and diagnostic cutoffs to maximize clinical utility. Longitudinal assessments must characterize VCAM-1 level dynamics throughout disease progression, clarifying their prognostic value to inform early intervention strategies, disease monitoring protocols, and therapeutic decision-making.

Advancing therapeutic strategies requires the development of novel pharmacological agents targeting VCAM-1. Priority should be given to developing highly specific VCAM-1 modulators through three principal approaches: small-molecule inhibitors targeting key structural domains, biologic agents with enhanced specificity, and gene-editing techniques for precise regulation of VCAM-1 expression. Combination therapeutic strategies warrant systematic investigation, with particular emphasis on examining potential synergies between VCAM-1-targeted interventions and established therapies such as Aβ-targeting agents, tau-directed treatments, and neuroprotective compounds. Furthermore, optimizing CNS drug delivery requires focused development of nanotechnology-based carriers including liposomes and polymeric nanoparticles, physical BBB modulation approaches such as focused ultrasound, and novel CNS-targeting formulations to achieve therapeutic drug concentrations in the brain parenchyma.

While significant progress has been made in understanding VCAM-1's role in cognitive impairment, several critical knowledge gaps remain unresolved. Key unresolved issues include potential heterogeneity in VCAM-1 regulation across distinct cognitive impairment subtypes and how such differences could inform precision medicine approaches. Additional critical questions concern the precise nature of VCAM-1's interactions with other neurovascular unit components during disease progression, along with the complex crosstalk between VCAM-1-driven neuroinflammation and co-occurring pathologies including oxidative stress and mitochondrial dysfunction, which collectively contribute to cognitive decline. Addressing these knowledge gaps will be crucial for developing more effective diagnostic tools and targeted therapies to improve clinical outcomes in cognitive impairment.
